# Optimal phylogenetic reconstruction of insertion and deletion events

**DOI:** 10.1093/bioinformatics/btae254

**Published:** 2024-06-28

**Authors:** Sanjana Tule, Gabriel Foley, Chongting Zhao, Michael Forbes, Mikael Bodén

**Affiliations:** School of Chemistry and Molecular Biosciences, The University of Queensland, Brisbane, QLD 4072, Australia; School of Chemistry and Molecular Biosciences, The University of Queensland, Brisbane, QLD 4072, Australia; School of Chemistry and Molecular Biosciences, The University of Queensland, Brisbane, QLD 4072, Australia; School of Mathematics and Physics, The University of Queensland, Brisbane, QLD 4072, Australia; School of Chemistry and Molecular Biosciences, The University of Queensland, Brisbane, QLD 4072, Australia

## Abstract

**Motivation:**

Insertions and deletions (indels) influence the genetic code in fundamentally distinct ways from substitutions, significantly impacting gene product structure and function. Despite their influence, the evolutionary history of indels is often neglected in phylogenetic tree inference and ancestral sequence reconstruction, hindering efforts to comprehend biological diversity determinants and engineer variants for medical and industrial applications.

**Results:**

We frame determining the optimal history of indel events as a single Mixed-Integer Programming (MIP) problem, across all branch points in a phylogenetic tree adhering to topological constraints, and all sites implied by a given set of aligned, extant sequences. By disentangling the impact on ancestral sequences at each branch point, this approach identifies the minimal indel events that jointly explain the diversity in sequences mapped to the tips of that tree. MIP can recover alternate optimal indel histories, if available. We evaluated MIP for indel inference on a dataset comprising 15 real phylogenetic trees associated with protein families ranging from 165 to 2000 extant sequences, and on 60 synthetic trees at comparable scales of data and reflecting realistic rates of mutation. Across relevant metrics, MIP outperformed alternative parsimony-based approaches and reported the fewest indel events, on par or below their occurrence in synthetic datasets. MIP offers a rational justification for indel patterns in extant sequences; importantly, it uniquely identifies global optima on complex protein data sets without making unrealistic assumptions of independence or evolutionary underpinnings, promising a deeper understanding of molecular evolution and aiding novel protein design.

**Availability and implementation:**

The implementation is available via GitHub at https://github.com/santule/indelmip.

## 1 Introduction

Insertions and deletions (indels) play a pivotal role in shaping the landscape of genetic diversity. The importance of indels in protein families lies in several key aspects: (i) indels can introduce variation in the length and composition of protein sequences, leading to anything from subtle to dramatic changes in protein structure and function, which in turn arm us to engineer novel proteins suited to medical and industrial applications (Shortle and Sondek 1995, Vetter *et al.* 1996, Kim and Guo 2010, Savino *et al.* 2022, Miton and Tokuriki 2023); (ii) indels serve as key evolutionary markers, offering insights into the history of and relationships among homologous sequences (Dessimoz and Gil 2010, Nagy *et al.* 2012, Luan *et al.* 2013); (iii) indels probe and inform the relationship between the multiple sequence alignment (MSA) and phylogenetic tree inference, since indels are much less homoplastic than substitutions (Lloyd and Calder 1991, Dwivedi and Gadagkar 2009). To ensure sequence characters line-up to indicate homology, the MSA uses gap characters to pad where deletions and insertions could have occurred in the evolutionary past, forming patterns that can only be resolved in relation to other included sequences.

A key difference between methods that account for or infer indels is how they represent their occurrence in extant sequences of a family, and then how they are encoded at ancestral branch points. That said, it is not unusual to simply treat indels as missing data (Yang 2007, Kozlov *et al.* 2019, Oliva *et al.* 2019).

A *position-specific* (PS) discrete encoding (often binary) assumes that (like any other character in an alignment) indels occupy a single site and does not allow indels to overlap or be subsets of other indels (Edwards and Shields 2004, Musil *et al.* 2020); this implies that calls of one site are independent of other sites in a molecular sequence, so finding the position-specific optimum is part of the global optimum (Thorne *et al.* 1991, 1992, Chindelevitch *et al.* 2006, Rivas and Eddy 2008, Bouchard-Côté and Jordan 2013). The problem with defining the global optimum this way is that both insertions and deletions can involve more than one consecutive position.

A *composite* discrete encoding, such as simple indel coding (SIC) (Simmons and Ochoterena 2000), represents *an indexed set of* indels that occur with different lengths and at different, potentially overlapping positions; with SIC, for any sequence across the input data, an indel is either present, absent, or inadmissible, which is used to flag when it is impossible to say, e.g. a deletion is precluded by an earlier, wider deletion (Simmons and Ochoterena 2000). A discrete state at each index (or position) lends itself in general to a multitude of inference algorithms, including maximum-likelihood (ML; provided a probabilistic model of transitions between the states) and maximum-parsimony. Indeed, FastML (Pupko *et al.* 2000) offers both for SIC, utilizing a gene loss/gain model for ML.

Partial order graphs (POGs) can represent a family of sequences via nodes for the *inclusion* of homologous content (using a global alignment index) and directed edges to selectively indicate the *exclusion* of content in other sequences (Grasso and Lee 2004). A single sequence can be generated by following edges from a global ‘dummy’ start to a ‘dummy’ end point; we consider each sequence as having an indel ‘footprint’ as represented by the set of edges that are followed. As in the ancestral sequence reconstruction method GRASP (Foley *et al.* 2022), we formulate the indel inference problem over the set of all edges. Each global alignment index is associated with a finite and discrete set of edges (each of which has a start and end index in turn), enabling the use of both ML and parsimony-based inference to choose which edge to traverse at each index.

In contrast to composite discrete encodings, edges can capture some positional dependencies, given that edges are chosen to the exclusivity of others that share one of the two alignment indices. Most existing indel inference methods solve a fragmented optimization problem, to be resolved at each internal branch point rather than a global problem across the phylogenetic tree. GRASP (Foley *et al.* 2022) uses a method referred to as bi-directional edge parsimony (BEP; or alternatively maximum-likelihood; BEML) that finds ‘optimal’ edges, but not optimal paths through them, failing to provide guarantees of the optimal composition of indels within each (ancestral) sequence; a solution is therefore not guaranteed to be globally optimal.

When indel dependencies and whole-sequence constraints are considered, the indel inference problem becomes non-trivial (Fredslund *et al.* 2003), and stymie probabilistic adaptations. Hence, we focus on parsimony-based formulations that do not account for evolutionary distances.

Fredslund and colleagues (Fredslund *et al.* 2003) address the presence of long indels (exceeding one site) by using the concept of a gap graph with an affine gap penalty function. Snir and Pachter (Snir and Pachter 2006) employ a dynamic programming approach to deduce the insertion and deletion history in long DNA sequences within the context of small species phylogenetic trees. The Snir and Pachter (SP) recursive dynamic programming method determines whether there is a gap or non-gap at the current position in the molecular sequence at a node. Reassuringly, it takes into account the previous position and the parent of the node being considered, but it comes with a time complexity exponential to the number of nodes in the tree, and this complexity is likely to hamper applications to protein datasets.

In this paper, we hypothesize that framing the indel inference problem as a single global optimization problem across all branch points in a phylogenetic tree accounting for multi-site dependencies amongst indels can lead to qualitatively and quantitatively better reconstruction of their evolutionary history relative to existing parsimony-based approaches.

We model indel inference in the phylogenetic tree as a maximum-parsimony problem using edges to explicitly represent multi-site dependencies amongst indels, as imprinted on the input sequence alignment when mapped to the phylogenetic tree. Our methodology draws inspiration from alignment algorithms, utilizing gap opening and pattern differences between two sequences as the global optimization objective. The constraints and objective function can be readily represented as a Mixed-Integer Programming (MIP) problem. The resulting MIP has a large embedded network flow component together with side constraints used to evaluate the objective. Our working hypothesis was that this MIP is tractable to solve in practice, even if it had millions of variables and constraints. Indel inference in phylogenetic tree is formally defined as a small parsimony problem with binary character states and classed as NP- or AJX-hard (Jin *et al.* 2009, Fischer *et al.* 2014, Scornavacca and Weller 2022).

Our assessment of indel solutions involves both quantitative and qualitative analyses, comparing them with other parsimony-based approaches based on three criteria: (i) total indel events, (ii) evolutionary cohesiveness, and (iii) indel footprints. For synthetic data, we are also able to compare the number of predicted indels to the actual number of indels introduced during evolution.

## 2 Materials and methods

### 2.1 Preliminaries

A phylogenetic tree T=(X,N,B) is a directed acyclic graph (DAG) that represents the evolutionary relationships *B* among a set of leaf (or extant) nodes *X* (indexing extant sequences in the alignment) and internal nodes (referred to as branch points) *N* (indexing the ancestors of *X*). Assuming we have *n* extant nodes then, as the tree is binary, we have *n* − 1 internal nodes. By convention we denote N={1,…,n − 1}, with 1 as the root node, and X={n,…,2n − 1}. For each branch (k,l) ∈ B (where *k* is the direct ancestor of *l*) we have k ∈ N, l ∈ X∪N and *k *<* l*. A multiple sequence alignment of extant nodes *X* is a mathematical representation of the arrangement of homologous molecular sequences from different species or sources. If the sequences are of length *m* we denote the sites in the sequences as S={1,…,m}. We encode the MSA using a matrix of binary data $A=a_{ki},k\;\in \;X,i\;\in \;S$ where 0 represents a gap and 1 any amino acid at the relevant site.

**Figure 1.**
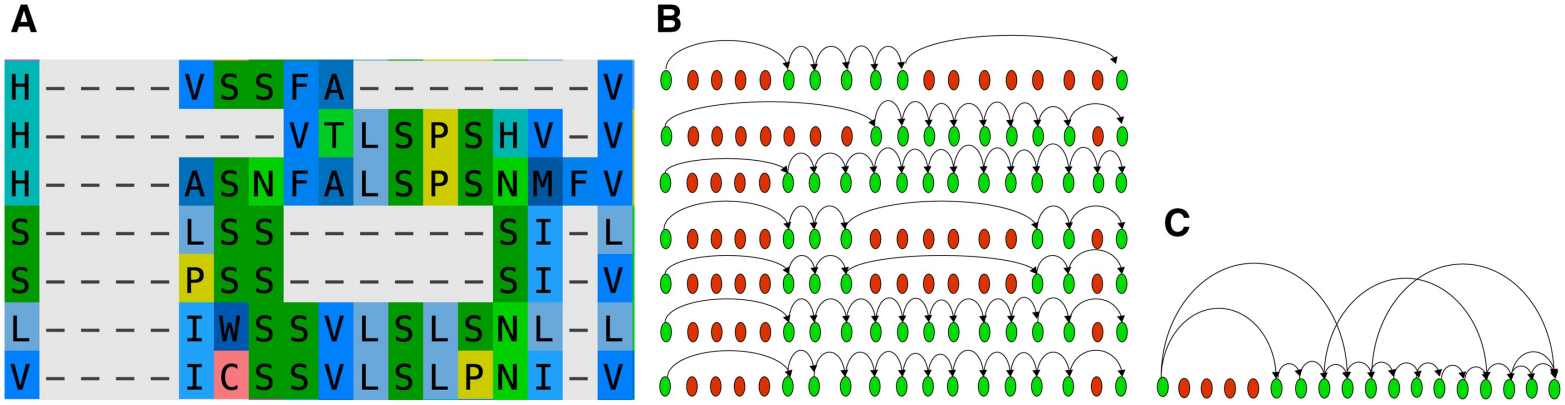
(A) An example MSA; (B) POGs from the example MSA, where green and red nodes represent non-gapped and gapped positions in a sequence, respectively; (C) Overall POAG representing the union of edges present in POGs.

We define *P*, a partial order alignment graph (POAG), as the union of the edges between adjacent occupied sites in *M*. More formally $P=\underset{k\;\in \;X}{\cup }\{(i,j)|i,j\;\in \;S,i\;<\;j,a_{ki}=1,a_{kj}=1,a_{ks}=0\forall i\;<\;s\;<\;j\}$. We denote the set of edges in *P* that arrive at site *i* as $P_{i}^{-}$ and the set of edges that leave site *i* as $P_{i}^{+}$. *P* captures the relationship between non-gapped positions across all sequences in the MSA, and we refer to this as the indel footprints in a given set of extant nodes *X*. Figure 1 shows a small MSA and its conversion to a POAG.

The first and last site in each sequence can be gapped, so we introduce ‘dummy’ start and end nodes in the POAG. We can, without loss of generality, preprocess our data to remove any consecutive sites that are gapped and any consecutive sites that are non-gapped in every sequence.

We focus on optimizing the indel events for a phylogenetic tree and an MSA using a MIP formulation where *T*, *A* and *P* are the input data structures. We compute the optimal path of edges forming a sequence for each of the nodes in *N* while restricting the indels used to those implied by the edges in *P*. For computing the optimal path, we use variables *d* as difference in position wise indel pattern and *g* as gap opening between two indel patterns.

The MIP uses the following decision variables:

-
$a_{ki}\;\in \;\{0,1\}$ encodes the gap or non-gap position at internal node k ∈ N at site i ∈ S. Note that *a_ki_* is input data for k ∈ X, but a variable for k ∈ N.
-
$y_{kp}\;\in \;\{0,1\}$ encodes the presence or absence of edge p ∈ P for internal node k ∈ N.
-
$d_{bi}\;\in \;\{0,1\}$ is 1 if the indel pattern is different for the two nodes in branch b ∈ B at site i ∈ S. These variables are used to count site differences between neighbours in *T*.
-
$g_{bi}\;\in \;\{0,1\}$ ie gap opening is 1 if a difference starts for the two nodes in branch b ∈ B at site i ∈ S. These variables are used to count the number of sequences of differences between neighbours in *T*.

We define MIP-Indel as:

(1) $$\min\sum_{b\;\in \;B}\sum_{i\;\in \;S}\left(d_{bi}\;+\;\alpha \cdot g_{bi}\right)$$

(2) $$\begin{array}{c} s.t.\sum_{p\;\in \;P_{i}^{-}}y_{kp}=a_{ki}\\ \;\;\;\;\;\;\;\;\;\;\;\;\forall i\;\in \;S,i\;>\;1,\forall k\;\in \;N \end{array}$$

(3) $$\begin{array}{c} \sum_{p\;\in \;P_{i}^{+}}y_{kp}=a_{ki}\\ \;\;\;\;\;\;\;\;\;\;\;\;\forall i\;\in \;S,i\;<\;s,\forall k\;\in \;N \end{array}$$

(4) $$\begin{array}{c} d_{(k,l)i}=\left|a_{ki}\;-\;a_{li}\right|\\ \;\;\;\;\;\;\;\;\;\;\;\;\forall i\;\in \;S,\forall (k,l)\;\in \;B \end{array}$$

(5) $$\begin{array}{c} g_{b1}=d_{b1}\\ \;\;\;\;\;\;\;\;\;\;\;\;\forall b\;\in \;B \end{array}$$

(6) $$\begin{array}{c} g_{bi}\;\geq \;d_{bi}\;-\;d_{b(i\;-\;1)}\\ \;\;\;\;\;\;\;\;\;\;\;\;\forall i\;\in \;S,i\;>\;1,\forall b\;\in \;B \end{array}$$

(7) $$\begin{array}{c} g_{(k,l)i}\;\geq \;a_{k(i\;-\;1)}\;+\;a_{li}\;+\;(1\;-\;a_{l(i\;-\;1)})\;+\;(1\;-\;a_{ki})\;-\;3\\ \;\;\;\;\;\;\;\;\;\;\;\;\forall i\;\in \;Si\;>\;1,\forall (k,l)\;\in \;B \end{array}$$

(8) $$\begin{array}{c} g_{(k,l)i}\;\geq \;a_{ki}\;+\;a_{l(i\;-\;1)}\;+\;(1\;-\;a_{li})\;+\;(1\;-\;a_{k(i\;-\;1)})\;-\;3\\ \;\;\;\;\;\;\;\;\;\;\;\;\forall i\;\in \;Si\;>\;1,\forall (k,l)\;\in \;B \end{array}$$

The objective value (1) minimizes a weighted combination of the number of individual site difference and the number of sequences of site differences. Constraints (2) and (3) ensure that site gap/non-gap indicator is equal to the total edges leaving and arriving at the site. Constraint (4) computes the site difference between the two ends of a branch in the tree. Constraints (5–8) compute the number of times sequence differences occur. Constraint (6) detects the beginning of series of differences—the sequences have been the same, but now they are different. Constraints (7) and (8) count the case where the sequences are different and stay different, but the gap opening patterns reverse. $\alpha \;\in \;R^{+}$ is the hyperparameter representing the importance of minimizing opening a new gap between indel patterns in two molecular sequences.

We offer a simplified example to illustrate the workings behind the previously defined constraints. Consider two sequences, E and F, with indel patterns represented as 10011 for E and 00101 for F, where E is the ancestor of F. The variable $d_{\left(E,F\right)}$ for all positions equals 10110 by taking position wise absolute differences. Correspondingly, the penalty term $g_{\left(E,F\right)}$ for all positions equals 10100 as per constraint 5 and 6. The penalty term for position one ie $g_{(E,F)1}\;\geq \;0\;-\;1=-1$. As a binary variable with positive objective coefficient in a minimization problem, it is set to 0. From (6) the penalty term for position four is $g_{(E,F)4}\;\geq \;1\;-\;1=0$. Position three represents an insertion of content in sequence F and position four a deletion of content in sequence F they would be treated as two discrete events unless penalized. Constraints (7) and (8) handle these special cases where gap patterns reverse. So, constraints (7) and (8) evaluate to $g_{(E,F)4}\;\geq \;\;-\;1$ and $g_{(E,F)4}\;\geq \;1$, which makes sure the $g_{(E,F)4}=1$ and is penalized.

### 2.2 Datasets

We determine the characteristics of predictions and computational performance of methods for indel inference using both real (but imperfectly aligned) protein families as well as sequence data that was generated by simulating protein evolution (resulting in perfect alignment). The latter provide us with opportunity to evaluate the ability of methods to reconstruct the synthetic history of mutations and determine the robustness at a greater level of granularity and control than is practical with biological data.

We use 15 real datasets from a varied range of protein families, with total extant sequences ranging from 165 to 2000 and aligned sequence length of protein sequences ranging between 513 and 5240. The real datasets are denoted by combining a brief protein family name with the total number of existing sequences. For instance, the dataset RNaseZ_624 comprises sequences of Metallo-*β*-lactamase family, encompassing a total of 624 protein sequences. For details on the datasets, please refer Supplementary Tables S1 and S2.

For synthetic datasets, we generate trees for n ∈ {300,500,700,1000,2000} extant sequences with a mean evolutionary distance δ ∈ {0.02,0.5,0.8,1} from a single root using the tool TrAVIS, which forms part of the GRASP-suite (Foley *et al.* 2022). TrAVIS samples the Gamma distribution γ(κ,θ) (where κ ∈ {0.5,1,2} defines the shape and θ=0.2 the scale) to set distances on each branch (in turn normalized to the mean *δ*), bi-furcating each branch point until the specified number of sequences have been mapped as leaves. Details on synthetic data generation using TrAVIS is covered in Supplementary Section—TrAVIS synthetic data generation.

Synthetic datasets are named by concatenating *n*, *δ* and *κ*. For example, t300d0.02s1 means a tree with 300 leaves with a mean distance of 0.02 to the root, generated using a Gamma shape parameter of 1.

We also generate a set of synthetic data using an independent tool INDELible (Fletcher and Yang 2009) exploring different values of its parameters. We generate for n ∈ {500,2000} extant sequences with different indel rate ζ ∈ {0.001,0.06,0.09}, keeping alpha *ϰ* at 0.5. Synthetic datasets from INDELible are named by concatenating *n* and *ζ* and *ϰ*. For example, t500i0.001a0.5 means a tree with 500 leaves with an indel rate of 0.001 and alpha of 0.5.

## 3 Results

We compare our MIP Indel method with other methods for indel inference, including BEP, PSP (parsimony method based on PS indel encoding) and SICP (parsimony method based on SIC indel encoding) (Pupko *et al.* 2000). We also compare against Snir and Pachter’s method that (similar to our approach) uniquely attempts to identify a globally optimal parsimonious solution.

The MIP model is solved using Gurobi (Gurobi Optimization, LLC 2023), the BEP, SICP, PSP are run using GRASP (Foley *et al.* 2022). For MIP, the value of hyperparameter *α* (to weight gap introduction and extension) is set to 2. Investigating the hyperparameter *α* reveals that the indel score typically diminishes with increasing *α*, eventually reaching a plateau (see Supplementary Fig. S6 for an exploration of *α* settings).

For BEP, SICP and PSP, we use the sequence (path through a POG) that GRASP produces by default for each branch point composed from multiple optimal solutions. In turn, this implies that indel events between ancestor sequences can originate from different (equally optimal) solutions for the same input data.

All the experiments were run on Dell PowerEdge R630 2 X 2.6 GHz 14-core Xeon (28 T) with 256 GB RAM, using a single thread with one hour total time limit per dataset. We attempted to make inferences on 15 real and 60 synthetic datasets; most methods returned solutions, but the SP method scaled poorly with tree size and was unable to complete analysis on any real data set.

### 3.1 MIP indel infers the least number of indel events

Post-inference, each branch (k,l) ∈ B (where *k* is the direct ancestor of *l*) implies a minimum number of indel events. Equation (9) breaks down the binary differences in (continuous) gap states between *k* and *l*.

(9) $$IE(l,i)=\{\begin{array}{cc} 1 & \text{if}\;a_{ki}\neq a_{li}\wedge (a_{ki\;-\;1}\neq a_{ki}\vee a_{li\;-\;1}\neq a_{li})\\ & \text{where}\;\;\exists k\;\in \;N,(k,l)\;\in \;B\\ 0 & \text{otherwise}. \end{array}$$

The indel score across the tree is defined by Equation (10).

(10) $$IS(T)=\sum_{l\;\in \;X}\sum_{i\;\in \;S}IE(l,i)\;+\;\sum_{k\;\in \;N}\sum_{i\;\in \;S}IE(k,i)$$

**Figure 2.**
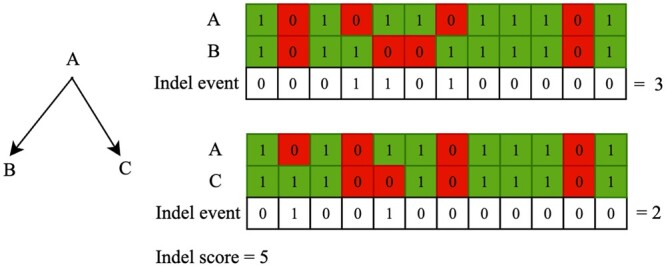
Example of indel events and score for an example tree. A is ancestor of B and C. 1 refers to a non-gap and 0 refers to gap at a site in a sequence alignment.

Figure 2 shows an example of how indel events are determined and how the indel score is calculated for a minimal example tree.

**Figure 3.**
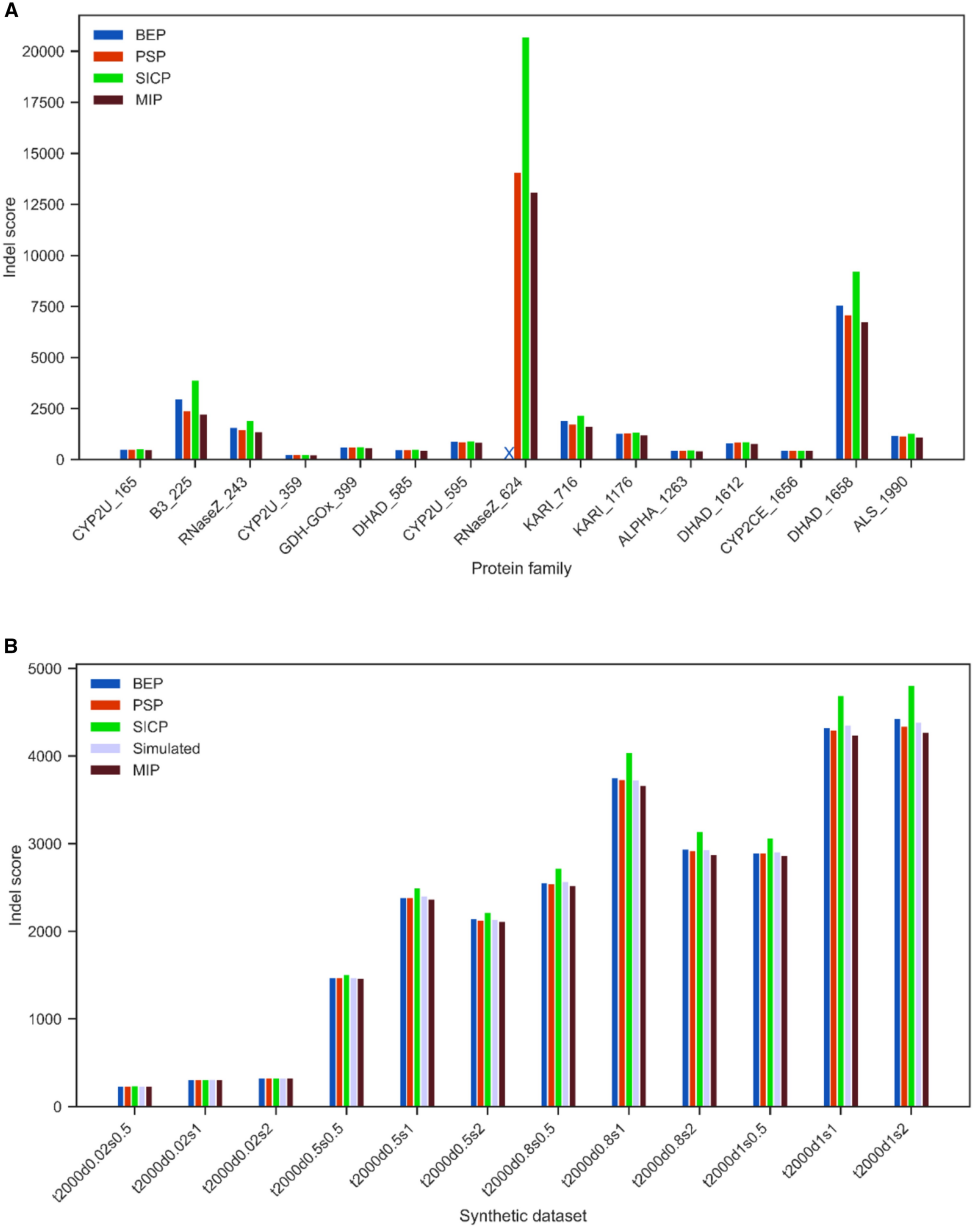
(A) Indel score for 15 real datasets. X in the above figure denotes that BEP did not provide a solution for the dataset RNaseZ_624; (B) Indel score comparison between (synthetic) ground truth and MIP Indel solution for trees with 2000 extant sequences with varying mean distance *δ* and *κ.*

The indel score for the 15 real datasets are shown in Fig. 3A. In all datasets, MIP Indel reports least indel score, followed by PSP, BEP and SICP methods. The RNaseZ_624 dataset represents a large, ancient protein family and commensurate sequence divergence is particularly challenging; for this data, all methods reported the greatest number of indels, and the BEP method failed to return any results within the time limit. The DHAD_1658 dataset is slightly less complex, and recapitulated the overall trend that MIP finds a solution with the smallest number of insertions and deletions, ahead of baseline position-specific (maximum) parsimony. Results for synthetic datasets in general corroborate the trends for the real data (see Supplementary Fig. S2).

We also compared MIP with the SP method on the smallest synthetic datasets (see Supplementary Fig. S7 on how these small synthetic datasets are generated). The SP method is unable to finish analysis on any of the other realistically large datasets; when completed, SP identified an equal or slightly greater number of indel events (see Supplementary Fig. S7).

For the 60 synthetic datasets, we have access to the intermediate, ancestral sequences. From these sequences, we derive indel patterns by assigning the values of 1 and 0 to gap and non-gap positions, respectively. Subsequently, employing the methodology outlined in Equations 9 and 10, we calculate the ‘ground truth’ indel score. Figure 3B presents a comparative visualization of the ground truth indel score alongside the indel scores obtained through various methods for distinct trees featuring 2000 extant sequences.

In instances where the mean distance within the tree is minimal, the MIP indel score strongly correlates with the ground truth indel score. Conversely, as the mean distances become larger, the ground truth score exceeds the MIP indel score. This outcome is anticipated, as greater distances elevate the likelihood of greater number of indel events between branch points. On the other hand, the SICP method scores higher than the ground truth as the mean distances become larger. Both BEP and PSP solutions closely align with the ground truth, with BEP method occasionally scoring higher than the ground truth.

**Figure 4.**
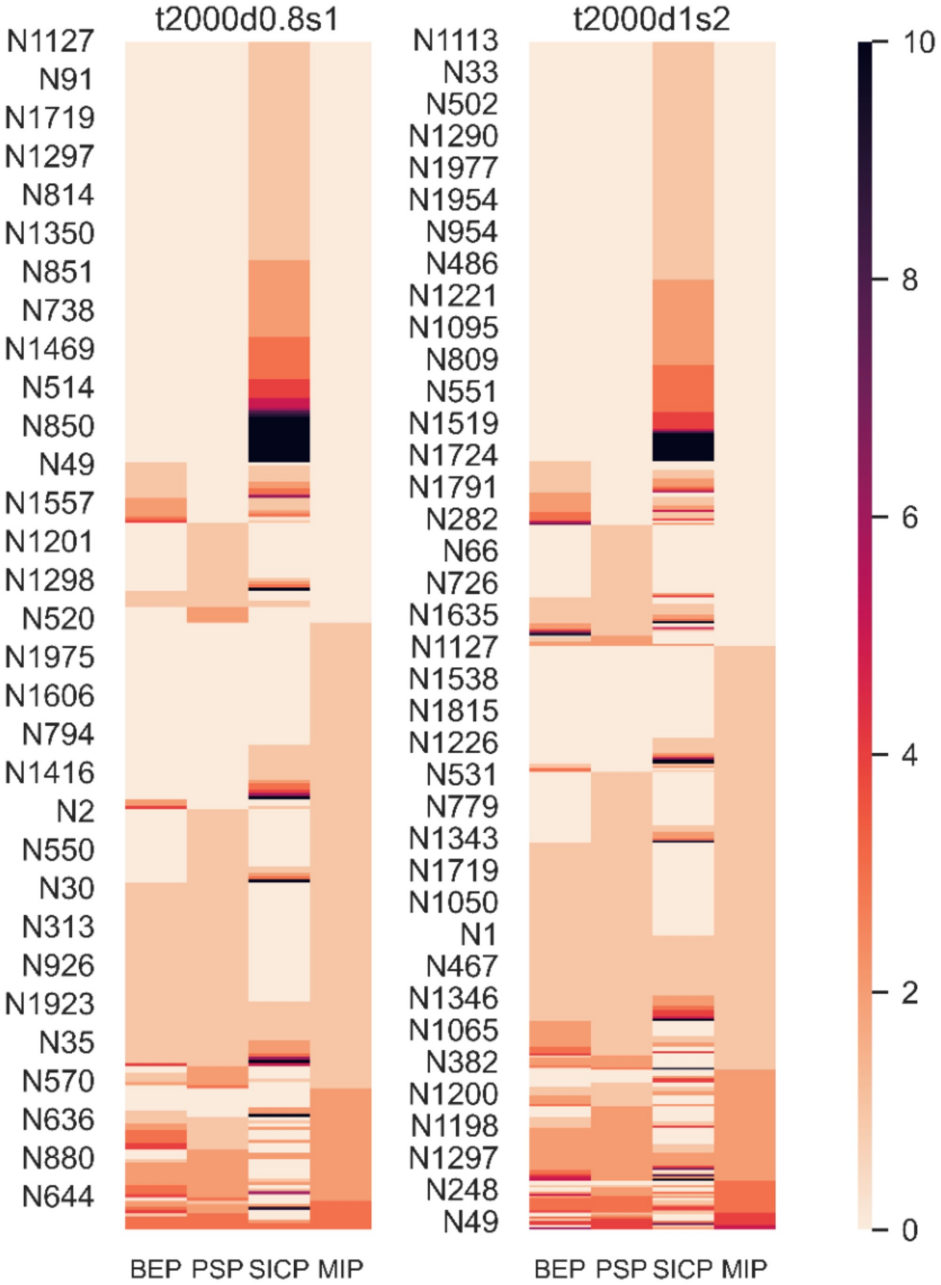
Comparison of ancestral indel patterns with synthetic ‘ground truth’ indel patterns (from TrAVIS) for trees t2000d0.8s1 and t2000d1s2. The ancestral nodes having no change in all four methods are not shown. The ancestral nodes are sorted in ascending order by number of pattern changes in MIP, PSP, BEP and SICP. The heatmap colourbar is scaled to a maximum value of 10.

The synthetic datasets contain a perfect record of indel states at all ancestors, and here we compare the four methods’ ability to reconstruct them for the synthetic datasets t2000d0.8s1 and t2000d1s2. When a method fails on a sequence, we also note the number of inaccurately placed indels, as illustrated in Fig. 4. When they fail, both BEP and SICP are further away from the correct indel patterns, relative to PSP and MIP, which reproduce ancestral indel patterns that are closer to those of the ground truth. However, for the 2000–2001 ancestors in each dataset, MIP reproduced fewer entirely correct indel patterns (1810 and 1725, respectively) than both PSP (at 1846 and 1735) and BEP (at 1878 and 1782; SICP performed worst overall with 1784 and 1653). An additional visualization for real dataset RNaseZ_243 is available in Supplementary Fig. S1.

Results from INDELible synthetic data are shown in Supplementary Fig. S5.

### 3.2 MIP indel solutions are evolutionarily cohesive

In contrast to methods that do not determine global optimas, and methods that assume degrees of site independence, MIP offers the distinct advantage in enforcing decisions that rationalize the *global* history of events. Across a tree there are scenarios of insertions and deletions that should not occur at all, e.g. a deletion event at a position that was inserted in an ancestor and subsequently deleted in its descendants. Additionally, there are mutational events that are highly unlikely to occur, e.g. a deletion occurring at the identical position in branch points directly descending from a common ancestor where content was inserted from its own ancestor.

**Figure 5.**
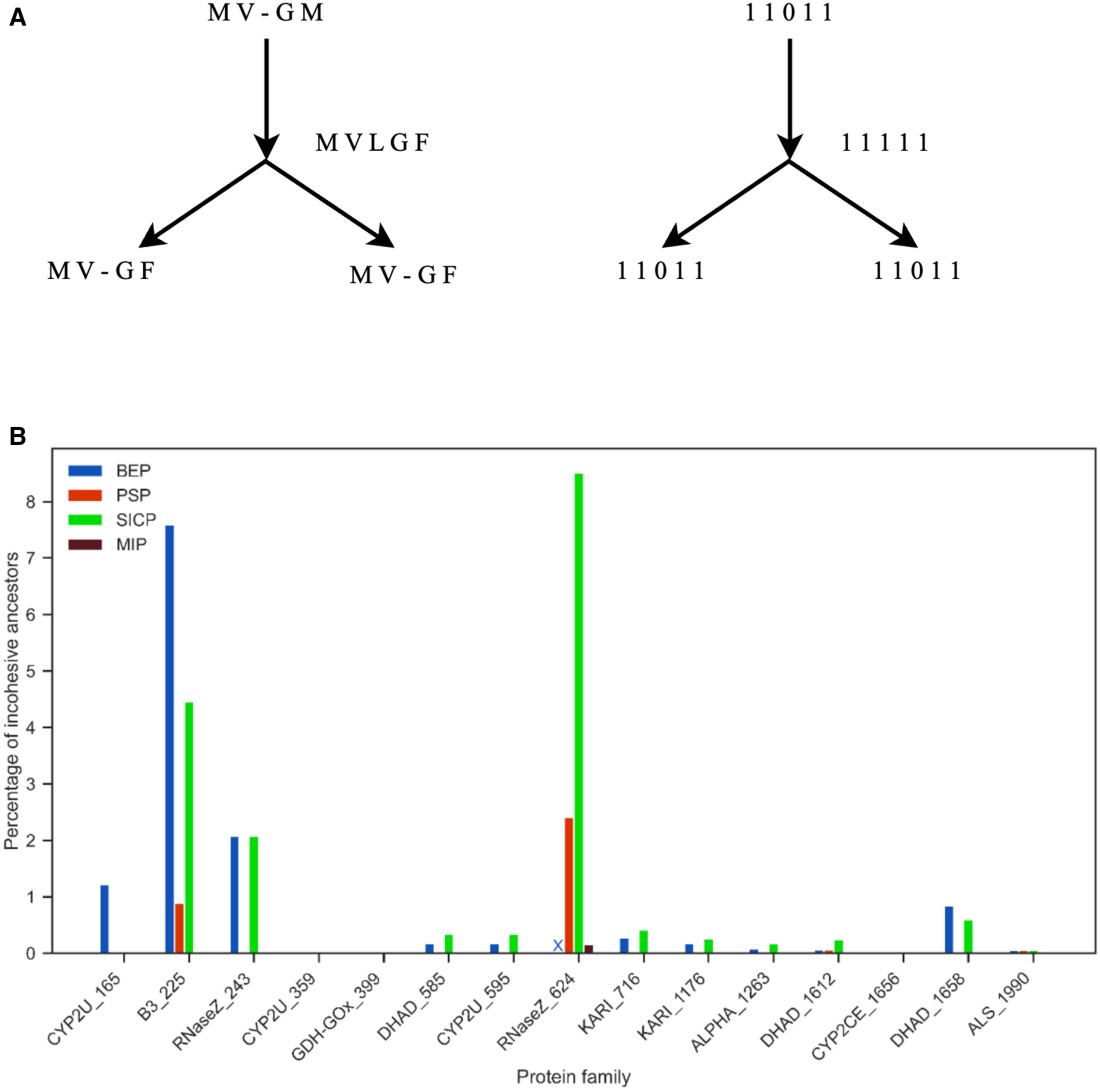
(A) Example of ‘incohesive’ indel states at third site for a sequence MVLGF at an ancestral branch point, where the indel state is distinct from both its descendants and its ancestor. (B) Percentage of incohesive ancestors for 15 real datasets. The symbol ‘X’ in the figure indicates that BEP did not find solution for the RNaseZ_624 dataset.

We define evolutionary cohesiveness by observing the changes in indel states *between* branch points. We quantify the number of branch points possessing at least one site where the predicted indel state differs completely from that of all its neighbours in the tree; specifically, a site being gapped at the branch point, when all its descendants and its ancestor are non-gapped, and vice versa. Illustrative scenarios are presented in Fig. 5A, where 0 and 1 represent a gapped and non-gapped state at a sequence site, respectively.

Biologically, these represent extremely unlikely events indicative of an interchanging history where either (i) sequence content is deleted and then immediately reinserted into the two children, or (ii) sequence content appears for one generation and then is immediately deleted in the two children. In the second scenario, this sequence content would also appear at other points in the tree, but this is also unlikely as it indicates separate independent insertions of the same content. We hypothesized that the level of cohesiveness of MIP indicates greater level of homogeneity relative to alternative methods. Indeed, MIP infers *no* such ancestors across all real and synthetic datasets (see Fig. 5B), except for one site involving one ancestor in the case of RNaseZ_624.

Both BEP and SICP methods fall short in cohesiveness for certain datasets, which is unsurprising given that they optimize indel events locally and/or do not account for dependencies. PSP appears to introduce a small number of incohesive branch points, but we explain this by the occurrence of multiple (equally optimal) solutions and that the implementation we use mixes how they are used across different ancestors. We expect that if we stopped at a single optimal solution, the column-specific cohesiveness for BEP and SICP would be improved slightly and be perfect in the case of PSP. Results for synthetic datasets corroborate the trends above (see Supplementary Fig. S3).

### 3.3 MIP indel solutions are constrained by the indel footprint in extants

Both MIP and BEP find solutions that are based on edges in the POAG, essentially constraining ancestors to exhibit combinations of the same indel patterns that are appear in one or more extant sequences. SICP and PSP performs inference at a different level of resolution; PSP uses patterns of individual sites present in extant sequences as input, which allow novel indel patterns to occur in ancestors once recast in terms of sequences (which in turn can be recast as edges forming part of a ancestral POAG). Analogously, SICP identifies a set of indel states each of which is inferred independently; their overlap can give rise to novel patterns. Here, we wanted to establish how methods compared in terms of how different ancestors looked relative to extant sequences.

**Figure 6.**
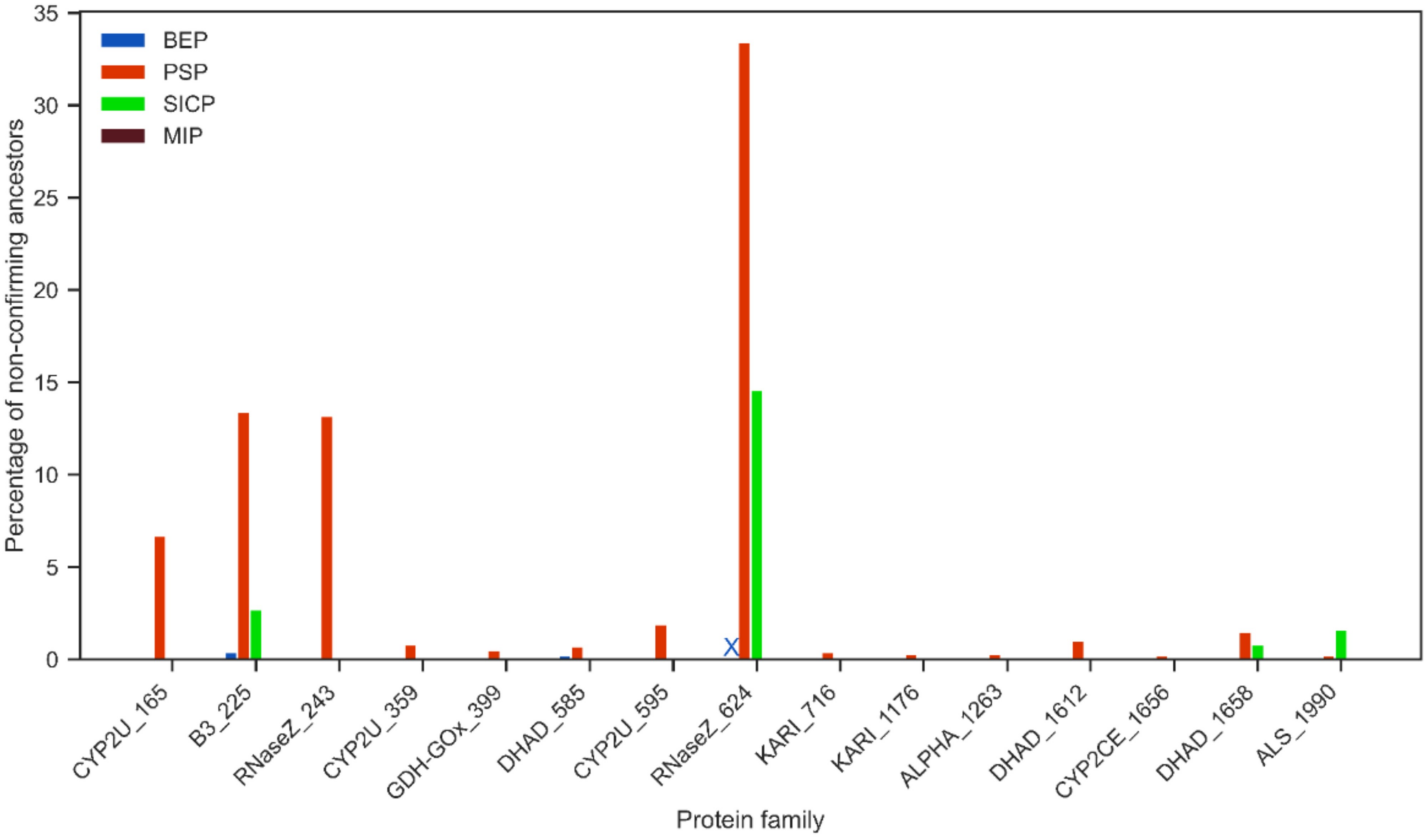
Percentage of non-conforming ancestors for 15 real datasets. X in the above figure denotes that a solution was not found.

For MIP, the inferred indel edges at an ancestral branch point constitute a subset of the edges present in the input POAG. This constraint ensures that the indel pattern inferred for an ancestor matches the indel footprints observed in the extant sequences. To quantify the degree of a match or (conversely) the distinctiveness of indels at ancestors, we counted ancestral branch points with ‘new’ edges across all datasets (see Fig. 6). Unsurprisingly, PSP creates the greatest number of ancestors with novel indel patterns across almost all datasets. SICP is much less creative but forms novel ancestral indels for the most complex datasets.

### 3.4 MIP indel typically finds alternative optimal indel histories

BEP is able to recover multiple indel histories that explain the same input equally well. The availability of indel options can be used by experimentally resurrecting distinct mutants likely to be structurally and functionally viable as demonstrated in (Foley *et al.* 2022). To investigate the hypothesis that phylogenetic trees encompass ambiguous optimal indel histories, we extend the mathematical MIP formulation (Section 2.1). This extension incorporates an additional constraint (Equation 11), to eliminate the initial optimal solution, allowing the MIP model to identify alternative optimal solutions.

(11) $$\sum_{n\;\in \;N}\sum_{i\;\in \;S}\sum_{p\;\in \;P_{i}^{-}|y_{\mathrm{nip}}^{*}=1}y_{\mathrm{nip}}\;\leq \;Q\;-\;1$$

where $y_{\mathrm{nip}}^{*}$ is the first optimal solution and $Q=\sum_{n\;\in \;N}\sum_{i\;\in \;S}\sum_{p\;\in \;P_{i}^{-}}y_{\mathrm{nip}}^{*}$ is the number of edges present in the first optimal solution. This prohibits the current solution, and only the current solution.

**Figure 7.**
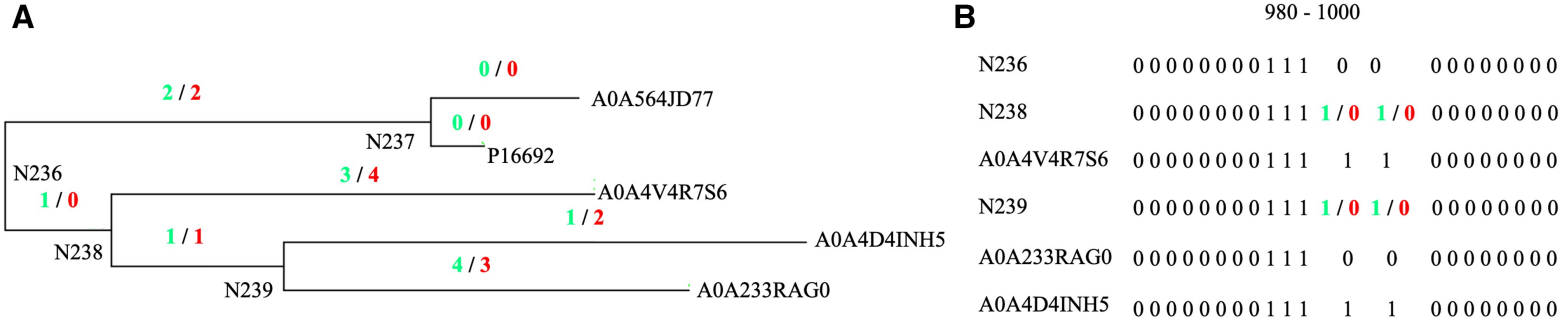
(A) The indel events at each branch point for optimal solution one and two (relative to the ancestor) is shown in green and red, respectively. Names starting with N are the ancestral branch point. We note that the total indel events in clade under ancestral branch point N236 is the same in both optimal solutions. The extants are represented by their UniProt identifiers. (B) Visualization of molecular sequence indel patterns for select nodes under the ancestral branch point N236 for sites from 980 to 1000. Ancestral branch point N237 is not shown as it has the same indel pattern in both optimal solutions. The sites, 991 and 992, accommodates both gap and ungap scenarios in a parsimonious manner. This is evident by the same sites in the extant sequences A0A233RAG0, A0A4D4INH5 and A0A4V4R7S6 present under the ancestral branch point of N238 and N239.

Out of 15 real datasets, MIP found alternate optimal indel histories for 12 datasets. We investigate the two alternative histories for RNaseZ_243 dataset and examine the indel events in the clade under the ancestral branch point N236 for the RNaseZ_243 tree across both optimal solutions. Fig. 7A shows the indel events for the clades under ancestral node N236. While the total number of indel events in the clade under N236 is consistent across both solutions, differences in indel patterns are observed at ancestral branch points N238 and N239 at sites 991 and 992, as illustrated in Fig. 7B.

## 4 Discussion

Indels factor heavily into the adaptive evolution of proteins. Yet, efforts to infer them—some based on evolutionary models (Rivas and Eddy 2008) and some accounting for their variable-length sequence imprint (Snir and Pachter 2006)—have had limited success in analysing realistically large protein families. The need to discern indel patterns in extant sequences and decode their history is transformed here into a combinatorial but practical optimization problem.

Mixed-Integer programming models are ideal for problems that involve identifying the optimal subset of items (decisions, activities) meeting specific criteria from a well-defined finite set of alternatives. Indel inference in phylogenetic trees, when framed as a MIP problem, offers many advantages.

A key benefit of our MIP formulation lies in treating indel inference as a *single, global* optimization problem. Reassuringly, the MIP approach has the same indel score as other parsimony-based methods for simple and small trees. This is particularly clear in the case of simulated data, where indel histories are well-defined and are not confounded by the sequence alignment step required for real data. Indeed, for simulated data, MIP always finds a solution that is equal or more parsimonious than the true history (which can contain unobservable, composite indel events).

Differences in indel solutions between methods emerge with larger trees, with the MIP approach consistently scoring conservatively lower on indels. MIP preserves the overarching connectedness of the tree, as evidenced by the quality of solutions. We note that comparable methods are led to local forms of optima and show that their combinations sometimes compromise cohesion of indel events when viewed across the whole tree.

The inclusion of constraints in the MIP model plays a crucial role in ensuring plausible histories. For instance, constraints restrict indels at internal branch points from deviating from their neighbours. Inspired by pairwise alignment, the objective function imposes (user-specified) affine gap penalties between ancestors and their direct descendants.

We also demonstrate that by formulating constraints around edges in a partial-order graph, the indel events at each ancestor do not deviate from those implied by extant sequences; the edges used to define each ancestor are a simple subset of those that define the input alignment.

The input does not always offer sufficient material to disambiguate solutions; we show MIP can report all indel events that explain the provided evidence. We expect this flexibility can be further harnessed to improve the quality of the underlying MSA, but also help explore diverse sequences and their histories for protein engineering.

Finally, it is worth noting that the MIP approach we present here is the first indel inference method that guarantees a globally optimal solution to realistically large protein family trees *in practice*; it is applicable to data of the scale that will support a wide range of analyses, e.g. to understand evolutionary drivers of antibiotic resistance and the engineering of enzymes for biofuel production.

Our benchmarking indicates that MIP Indel inference succeeds in finding optimal solutions for trees encompassing over 2000 extant proteins in reasonable time frame on modest hardware. This efficiency stands in stark contrast to other global optimization methods like SP, where it is impractical to find solutions for trees with as few as 30 extant proteins. However MIP’s scalability can exhibit exponential growth in worst-case schenarios, leading to deteriorating solving capabilities for large trees. Trees with 10,000 sequences are unlikely to finish within a few hours without heuristics, which in turn compromises the attractive guarantees of optimality.

## Supplementary Material

btae254_Supplementary_Data
