# Supplementary material for: Optimal phylogenetic reconstruction of insertion and deletion events
Source: Bioinformatics. 2024 Jun 28;40(Suppl 1):i277–86. doi: 10.1093/bioinformatics/btae254 (PMC11211827; doi:10.1093/bioinformatics/btae254)
Supplement: btae254_Supplementary_Data [file btae254_supplementary_data.pdf]

Supplementary Material

The details of the real datasets used in this paper are shown in Table S1.

The dataset CYP_165, CYP_359, GDH-GOx_399, DHAD_585, CYP_595, KARI_1176, DHAD_1612 are referenced from Foley et al. (2022). The details for the curation of the datasets B3_225, RNaseZ_243, RNaseZ_624, KARI_716, ALPHA_1263, CYP2CE_1656, DHAD_1658 and ALS_1990 is described in the Table S2. In all the datasets, the sequences were aligned using MAFFT and trees were inferred using IQTREE2.

TrAVIS synthetic data generation

This section outlines the details around how synthetic data is generated using TrAVIS, which forms part of the GRASP-suite Foley et al. (2022). TrAVIS samples the Gamma distribution $\gamma(\kappa, \theta)$ (where $\kappa \in \{0.5, 1, 2\}$ defines the shape and $\theta = 0.2$ the scale) to set distances on each branch (in turn normalised to the mean δ), bi-furcating each branch point until the specified number of sequences have been mapped as leaves.

For each tree, substitutions, insertions and deletions were randomly introduced at each branch point. Starting at the root with an arbitrary amino acid sequence with a length 80 or longer, the sequence at each of its children is determined recursively. For a sequence, each position is considered as a possible site for a mutation as a Poisson process with a relative rate $2r$ ($r = 1$ for all our synthetic data); following the recipe given by Cartwright Cartwright (2008), $\eta = e^{-2rt}$ is the probability that no indel has occurred, which implies a substitution (determined via a model like that suggested by Le and Gasquel Le and Gascuel (2008), for instance); the probability of an insertion is $(1 - \eta)/2$ which is equivalent to that of a deletion. Finally, the length of the insertion (or deletion) is given by a sampling the Poisson distribution $f(\lambda)$ (where mean $\lambda = 1$, which will give 0 or 1 37% of the time, with greater widths less frequent). Depending on the length of the sequence provided at the root, the resulting aligned sequences ranged from 143 to 2606 positions across the 60 trees.

Visualisation of indel events

We were curious to understand how methods differ qualitatively, including how indel events distribute across trees. We developed a visualisation where the root node is designated as having zero indels. Following a post-order traversal of the tree structure, we enumerate subsequent events in relation to the root node and cumulatively show their number on the y-axis. The x-axis signifies the cumulative level of the internal node relative to the root at 0. We show the predicted indel events for RNaseZ_243 in Figure S1 illustrating no particular bias and the overall trend that MIP includes least events.

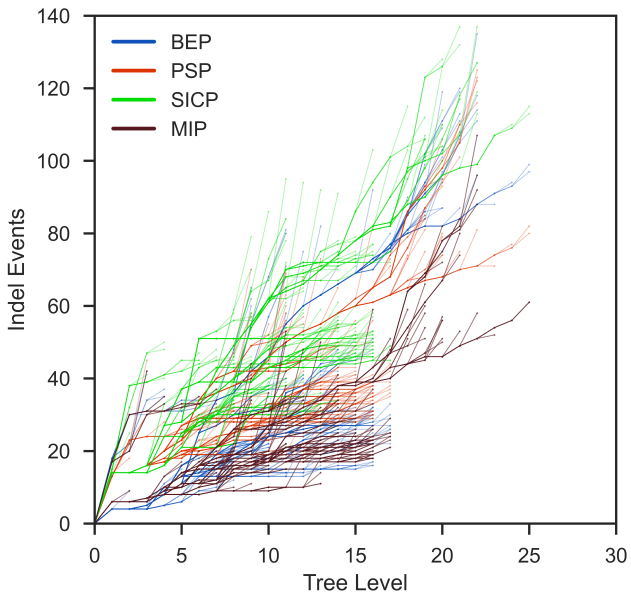

Fig. S1. Cumulative indel events predicted (y-axis) for the RNaseZ_243 dataset disperse evenly across different levels of the tree (x-axis) for all methods. MIP tends to predict fewer indels (brown lines).

Results on synthetic datasets

Indel scores for synthetic datasets

Figure S2 shows indel scores for 48 synthetic datasets.

Evolutionary cohesiveness in synthetic datasets

Figure S3 shows percentage of branch points where at least one indel state differs from all its descendants and ancestor in 48 synthetic datasets.

Indel footprints in synthetic datasets

Figure S4 shows percentage of non-confirming ancestors in 48 synthetic datasets.

Indel scores for INDELible synthetic datasets

Figure S5 shows indel scores for six synthetic datasets.

Table S1. Real protein families datasets used for MIP Indel evaluation.

Dataset	Protein family	Total extants	Aligned sequence length
CYP_165	Cytochrome P450	165	627
B3_225	Metallo-β-lactamase B3	225	1652
RNaseZ_243	Metallo-β-lactamase	243	1213
CYP_359	Cytochrome P450	359	542
GDH-GOx_399	Glutamate dehydrogenase	399	819
DHAD_585	Dihydroxy-acid dehydratase	585	776
CYP_595	Cytochrome P450	595	664
RNaseZ_624	Metallo-β-lactamase	624	5240
KARI_716	Ketol-acid reductoisomerase	716	735
KARI_1176	Ketol-acid reductoisomerase	1176	513
ALPHA_1263	Alphavirus	1263	1567
DHAD_1612	Dihydroxy-acid dehydratase	1612	835
CYP2CE_1656	Cytochrome P450	1656	1277
DHAD_1658	Dihydroxy-acid dehydratase	1658	2525
ALS_1990	Acetolactate synthase	1990	851

Table S2. Information on the remaining real datasets used in this paper (not referenced from Foley et al. (2022)). These datasets are newly created for different protein families.

Dataset	Detail
B3_225	The sequences belong to subclass B3 Metallo-β-lactamases. We use Glyoxalase II as an outgroup.
RNaseZ_243, RNaseZ_624	RNaseZ_243 and RNaseZ_624 are Ribonuclease Z sequences across archaea, bacteria, and eukaryotes either limiting the eukaryotic sequences to expected ortholog of the human elaC 1 protein (RNaseZ_243) or also including ortholog of the longer human elaC 2 (RNaseZ_624). We use PhnP sequences, a phosphodiesterase, as an outgroup.
KARI_716	The sequences belong to Class I ketol-acid reductoisomerases. We place the tree root on the branch between the archaeal and bacterial sequences.
ALPHA_1263	The sequences belong to 38 species and 1263 non-redundant sequences from the Alphavirus genus (Family Togaviridae). The tree is rooted at the midpoint.
CYP2CE_1656	The sequences belong to the Cytochrome P450 subfamilies CYP2C and CYP2E. We use vertebrate CYP2 family as an outgroup.
DHAD_1658	DHAD_1658 are dihydroxyacid dehydratases and sugar acid-specific dehydratases from the ilvD/EDD superfamily which includes representation of functionally-distinct clades. We use 6-Phosphogluconate dehydrogenase sequences as an outgroup.
ALS_1990	ALS_1990 are acetolactate synthases and we use a small selection of anabolic acetohydroxy-acid synthases as an outgroup.

Hyperparameter experiments

We conducted experiments to gauge the impact of hyperparameter α on indel scores for the synthetic dataset t300d0.5s0.5 (see Figure S6). The indel score peaks when α is zero since there is no penalty for gap opening. As α increases, the indel score decreases, and then levels off.

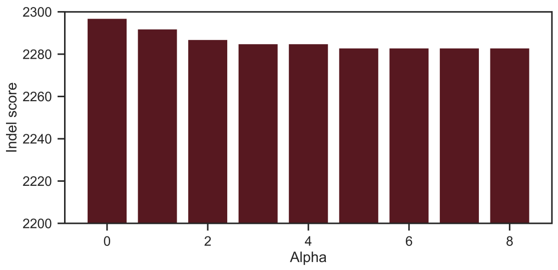

Fig. S6. Indel score for different α.

Comparison with Snir and Pachter's method.

Here we present the results of experiments from running indel inference on very small synthetic phylogenetic trees generated by tool TrAVIS using δ of 1.0 and κ of 1.1. We generate datasets by varying the number of extants and sequence positions, assigning them corresponding names. For example, the label `t10p100` denotes a tree with 10 leaves and sequences containing 100 positions. The MIP Indel inference method is compared against the dynamic programming method proposed by Snir and Pachter (2011). We implemented the SP algorithm and made it available at https://github.com/Tingzhaooo/spindel.

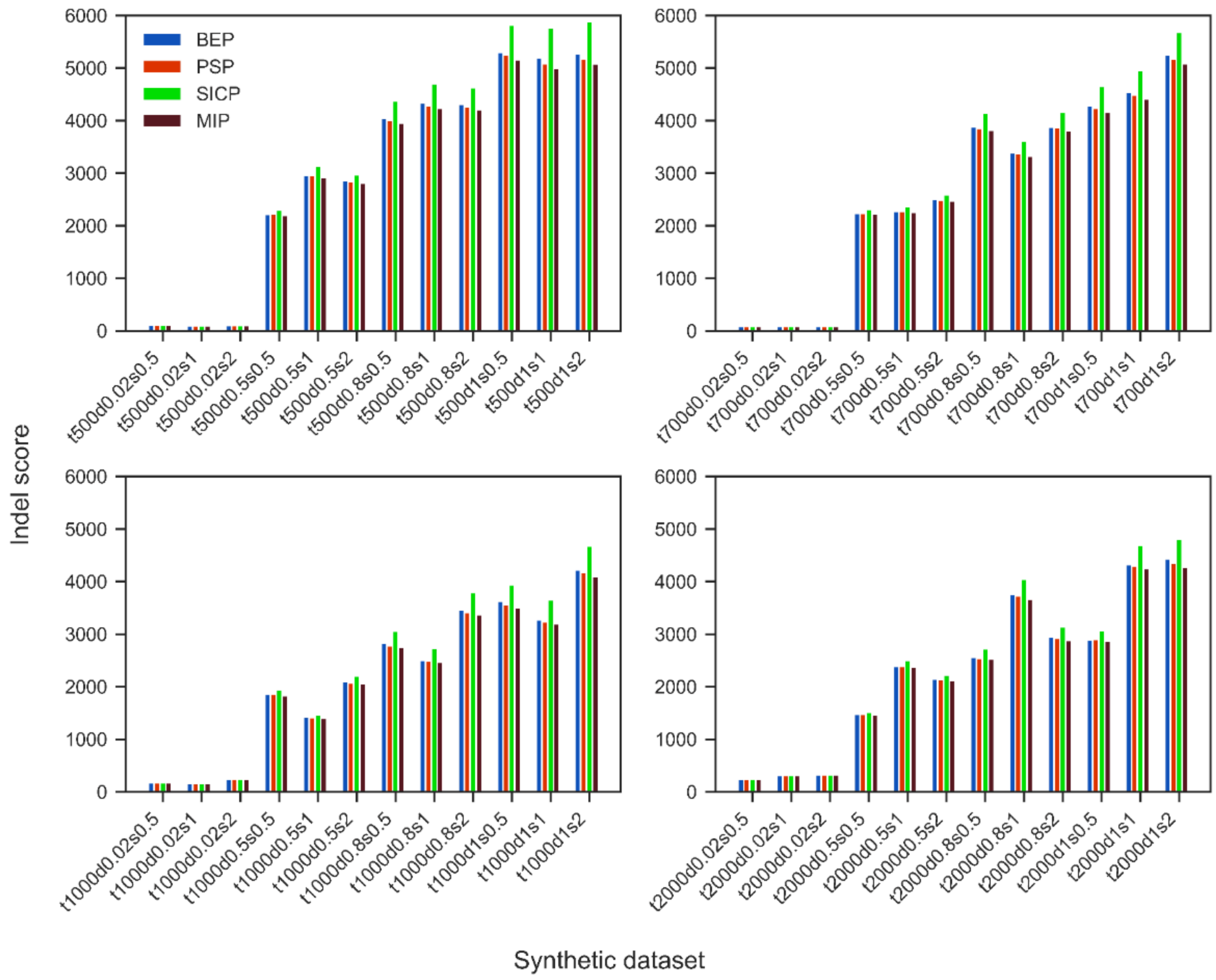

Fig. S2. Indel scores for 48 synthetic datasets. Remaining 12 not shown as they report similar trends.

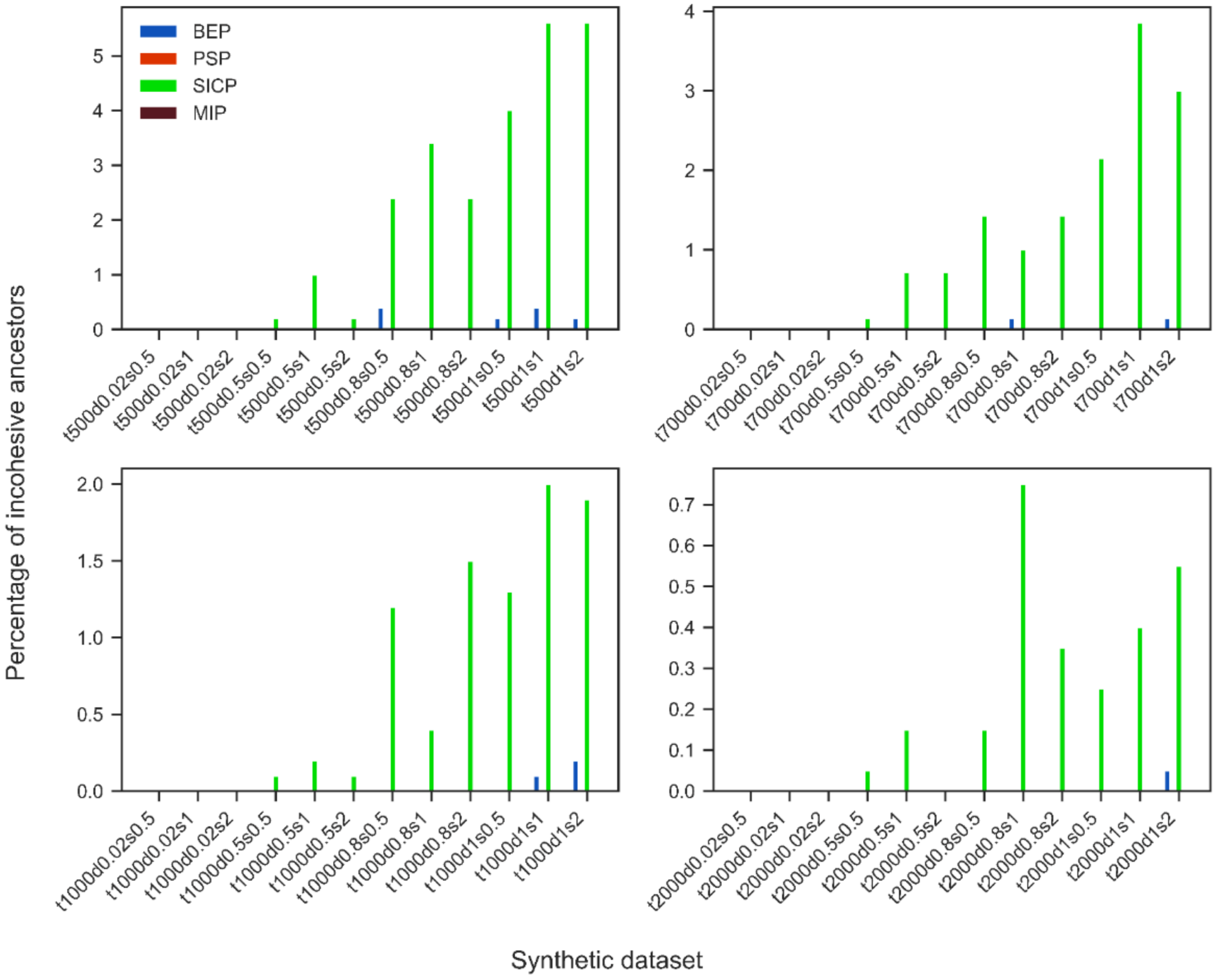

Fig. S3. Percentage of incohesive ancestors for 48 synthetic datasets. Remaining 12 not shown as they report similar trends.

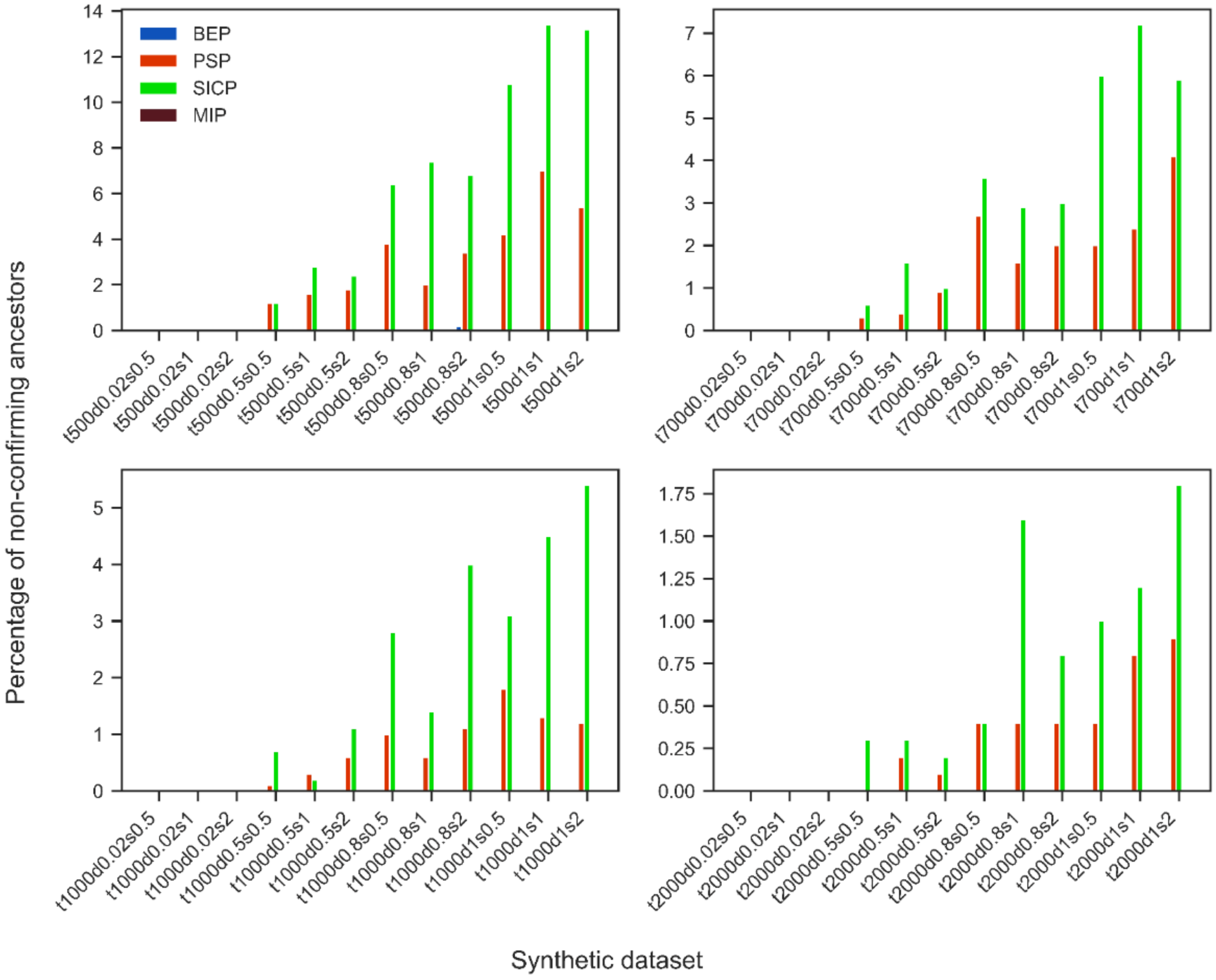

Fig. S4. Percentage of non-confirming ancestors for 48 synthetic datasets. Remaining 12 not shown as they report similar trends.

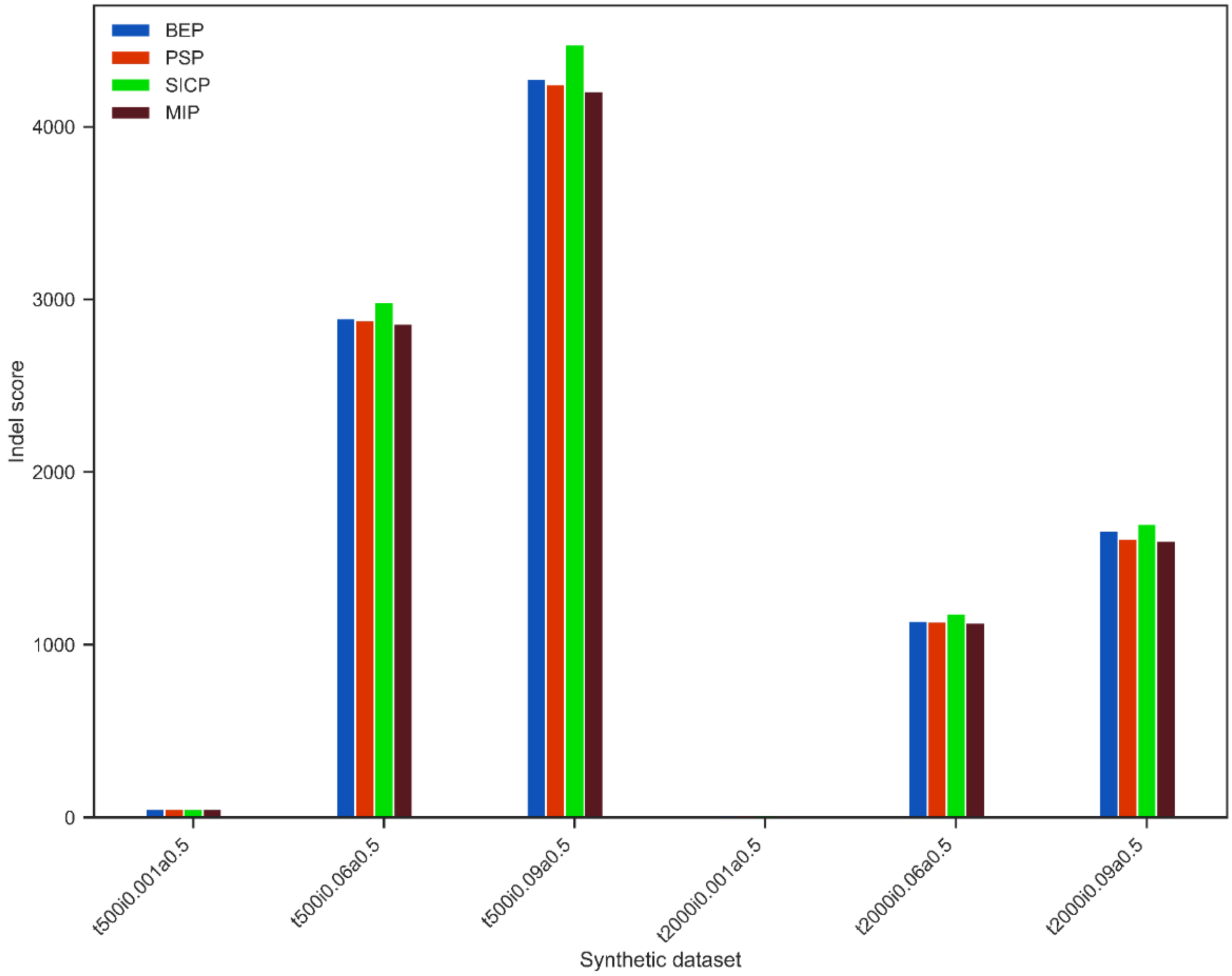

Fig. S5. Indel scores for six synthetic datasets generated from INDELible simulation tool.

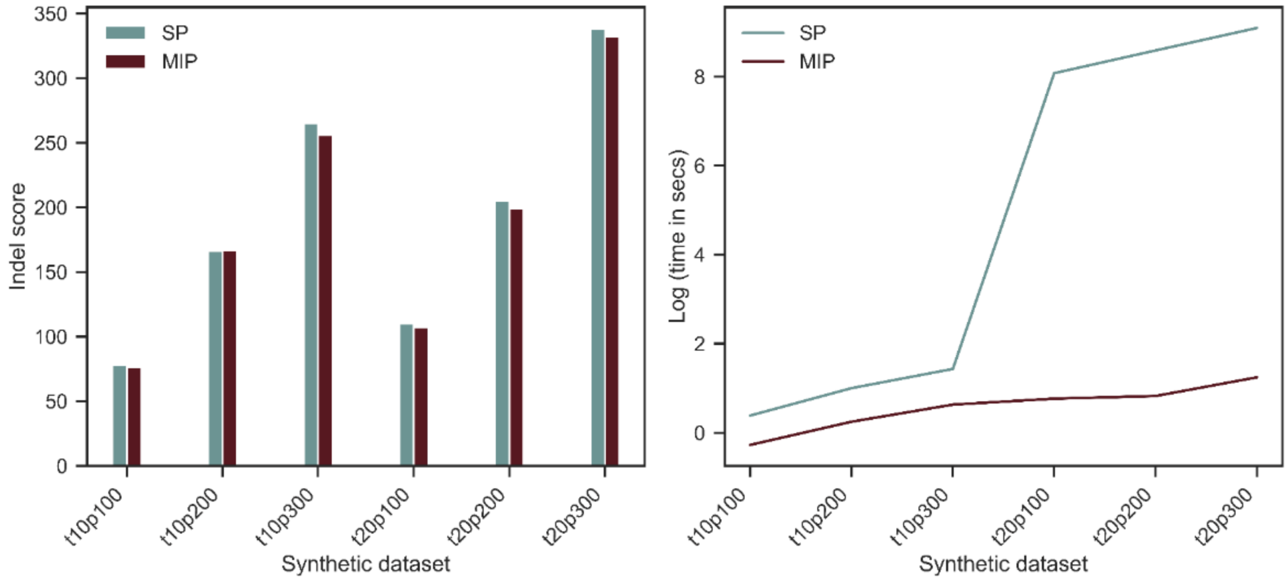

Fig. S7. Indel scores and time performance of Snir and Pachter's method (SP) compared with MIP.
